# MIRAGE syndrome caused by a novel missense variant (p.Ala1479Ser) in the *SAMD9* gene

**DOI:** 10.1038/s41439-020-0091-5

**Published:** 2020-03-05

**Authors:** Shinsuke Onuma, Tamaki Wada, Ryosuke Araki, Kazuko Wada, Kanako Tanase-Nakao, Satoshi Narumi, Miho Fukui, Yasuko Shoji, Yuri Etani, Shinobu Ida, Masanobu Kawai

**Affiliations:** 1 Department of Gastroenterology, Nutrition, and Endocrinology, Osaka Women’s and Children’s Hospital, Osaka, Japan; 2 Department of Neonatology, Osaka Women’s and Children’s Hospital, Osaka, Japan; 3 Department of Molecular Endocrinology, National Research Institute for Child Health and Development, Tokyo, Japan; 4 Department of Bone and Mineral Research, Research Institute, Osaka Women’s and Children’s Hospital, Osaka, Japan

**Keywords:** Adrenal gland diseases, Next-generation sequencing, Rare variants, Adrenal gland diseases, Next-generation sequencing

## Abstract

MIRAGE syndrome is a recently identified disorder characterized by myelodysplasia, infection, restriction of growth, adrenal hypoplasia, genital phenotypes, and enteropathy. It is caused by a gain-of-function variant in the *SAMD9* gene, but there is limited knowledge regarding the genotype–phenotype correlation. We herein report a Japanese patient with MIRAGE syndrome carrying a novel de novo heterozygous missense variant in the *SAMD9* gene (c.4435 G > T; p.Ala1479Ser).

MIRAGE syndrome (OMIM #617053) is a recently discovered disorder with multifaceted clinical features, including myelodysplasia, infection, restriction of growth, adrenal hypoplasia, genital phenotypes, and enteropathy^1^. Additional associated features are developmental retardation, dysmorphism, chronic lung disease, and central nervous system abnormalities. MIRAGE syndrome is caused by a gain-of-function variant in the *SAMD9* gene on the long arm of chromosome 7 (7q21.2), encoding sterile alpha motif domain-containing protein 9, which functions in endosome fusion and regulates cell growth in in vitro models^1,2^. However, the precise mechanisms by which variants in the *SAMD9* gene cause the MIRAGE phenotype are largely unknown. In addition, a wide range of phenotypes with a high mortality rate has been implicated with little understanding of the genotype–phenotype correlation due to the paucity of genetically diagnosed subjects. Therefore, the accumulation of patient-based knowledge with underlying genetic information is essential to better understand the clinical outcome, its relation to genotype, and molecular insights of the disorder. We herein present a case of MIRAGE syndrome with a novel de novo missense variant [NM_017654.4:c.4435 G > T, p.(Ala1479Ser)] in the *SAMD9* gene.

The 2-year-old patient was born from healthy nonconsanguineous Japanese parents. The family history was unremarkable, and his two older sisters are healthy. Fetal growth restriction was detected at 27 weeks of gestation by sonography. The patient was delivered at 31 weeks of gestation by cesarean section due to fetal distress. His birth weight was 912 g (−3.2 SD), length was 37.8 cm (−1.3 SD), and head circumference was 26.6 cm (−0.3 SD), based on the sex-specific standards stratified by gestational age in the Japanese population^3^. The Apgar scores were 7 and 8 at 1 and 5 min, respectively. He had ambiguous genitalia (micropenis, hypospadias, and bifid scrotum) without signs of female internal genitalia such as uterus and vagina at birth (Fig. 1a), and G-banding chromosome analysis revealed a 46, XY karyotype. Accordingly, he was assigned male. Monosomy 7 was not noted by G-banding chromosome analysis at birth. His skin was hyperpigmented (Fig. 1b). He was recognized as having adrenal insufficiency based on hormonal measurements (Table 1) and hypoplasia of the adrenal gland on abdominal sonography. Treatment with hydrocortisone (30 mg/m^2^/day) and fludrocortisone (0.025 mg/day) was initiated with subsequent dose adjustments being performed based on the clinical and laboratory findings. Transient thrombocytopenia and leucopenia were detected during the first 2 months of life, and there were no signs suggesting the presence of myelodysplastic syndrome (MDS) thereafter. Bone marrow biopsy was not performed. Macrocytosis was never found in the peripheral blood. As the patient developed intolerance to enteral feeding due to severe diarrhea, parenteral nutrition was initiated. He continues to receive total parenteral nutrition because resuming enteral nutrition reignites gastrointestinal symptoms. He was administered several cycles of antibiotics because of recurrent infections, including aspiration pneumonia. To prevent recurrent infections, continuous antibiotic prophylaxis with sulfamethoxazole-trimethoprim was initiated. He exhibited several episodes of acute adrenal crisis caused by acute infection and/or aspiration and received high doses of hydrocortisone (100 mg/m^2^/day). He developed hydrocephalus after the episode of adrenal crisis, and a ventriculoperitoneal shunt was placed at 7 months old. He had been repeatedly moved to the intensive care unit due to respiratory complications such as aspiration pneumonia, and a laryngotracheal separation procedure was performed at 2 years old. He has received continuous positive airway pressure (CPAP) support after laryngotracheal separation. He has never been discharged from the hospital since birth. His growth was markedly restricted during follow-up (Fig. 1c). He showed severe psychomotor developmental delay. He was bedridden and spoke no meaningful words at 2 years old. The presence of adrenal hypoplasia, ambiguous genitalia, transient thrombocytopenia, bowel dysfunction, recurrent infections, and failure to thrive are consistent with MIRAGE syndrome.

**Fig. 1:**
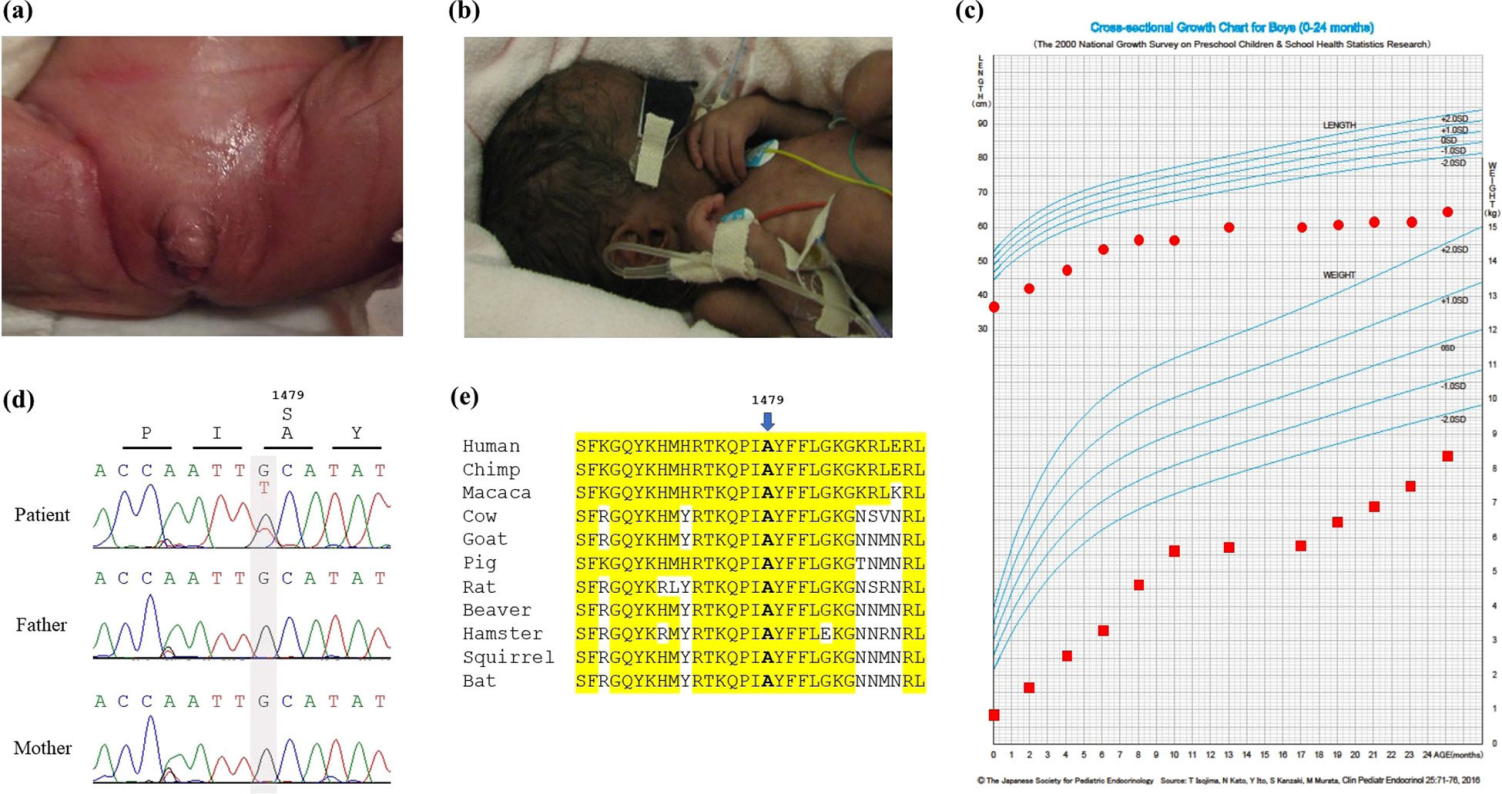
Clinical and genetic features of the patient. **a**, **b** Photographs of the patient at birth showing ambiguous genitalia (**a**) and hyperpigmentation (**b**). **c** Growth chart of the patient from 0 to 2 years old^19^. The red circle and square represent the patient’s height and weight, respectively. **d** Sanger sequences of the *SAMD9* gene in the patient and parents. A heterozygous de novo variant (c.4435 G > T; p.Ala1479Ser) was found in the patient. **e** Homologs of the *SAMD9* gene at the A1479 residue, which is conserved across multiple species.

**Table 1:** Laboratory data at birth.

Variables	Results	Reference values (mean ± 2SD)	Variables	Results	Reference values (mean ± 2SD)
WBC (×10^3^/µL)	2.9	19.6 ± 11.2	DHEA-S (µg/dL)	63	184 ± 104
Hb (g/dL)	18.1	19.0 ± 4.0	17-OHP (ng/mL)	1.97	4.5 ± 7.6
Plt (×10^4^/µL)	2.7	28.8 ± 10.6	AMH (ng/mL)	16.9	52.3 ± 47.4
Na (mEq/L)	138	143 ± 12	LH (mIU/mL)	0.9	0.39 ± 0.96
K (mEq/L)	4.1	5.3 ± 1.2	FSH (mIU/mL)	1.8	0.96 ± 1.20
BS (mg/dL)	56	63 ± 44	Testosterone (ng/dL)	175.3	85.9 ± 112.6
ACTH (pg/mL)	2540	50.2 ± 13.8	Estradiol (pg/mL)	<5	26.1 ± 77.8
Cortisol (µg/dL)	5.6	14.8 ± 3.8	TSH (µIU/mL)	4.89	8.4 ± 9.6
PRA (ng/mL/hr)	84.7	7.62 ± 13.58	FT4 (ng/dL)	0.89	1.00 ± 0.50
Aldosterone (pg/mL)	11.6	726 ± 570	FT3 (pg/mL)	0.87	1.04 ± 0.98

*PRA* plasma renin activity, *DHEA-S* dehydroepiandrosterone sulfate, *17-OHP* 17-hydroxyprogesterone, *AMH* anti-Müllerian hormone.

Values outside the reference ranges for neonates are underlined.

Written informed consent for genetic testing was received from the patient’s parents at 6 months of age. Long-range PCR-based targeted next-generation sequencing, as conducted previously^4^, revealed the presence of a heterozygous missense variant in exon 3 of the *SAMD9* gene [NM_017654.4:c.4435 G > T, p.(Ala1479Ser)]. This variant was confirmed by Sanger sequencing (Fig. 1d). Sanger sequencing of the region containing the identified variant revealed that this variant was not observed in the parents (Fig. 1d). No additional analyses were performed in parents. The identified variant was not observed in the variant database [gnomAD (https://gnomad.broadinstitute.org/), the 1000 Genomes database (https://www.internationalgenome.org/)], or the Human Gene Mutation Database (http://www.hgmd.cf.ac.uk/)]). For in silico analyses, the variant was predicted to be “damaging” with a score of 0.023 by SIFT and “possibly damaging” with a score of 0.689 by PolyPhen-2. This amino acid is conserved across the species evaluated^5^ (Fig. 1e). No variant was found in the PCNA-binding domain of the *CDKN1C* gene, based on which the diagnosis of IMAGe syndrome was excluded. Based on the ACMG/AMP standards and guidelines for the interpretation of sequence variants^6^, the identified variant was categorized as PS2, PM2, PP3, and PP4. Accordingly, we identified a likely pathogenic de novo variant in the *SAMD9* gene of a patient with MIRAGE syndrome.

We report a patient with MIRAGE syndrome who possesses a de novo novel missense variant in the *SAMD9* gene. Although we have not performed functional analysis of the identified variant, we believe it to be causative for MIRAGE syndrome based on the following findings: (1) the variant is rare based on the variant database, (2) the variant is damaging based on the in silico analyses, and (3) the mutated amino acid is relatively conserved among several species. As there is evidence demonstrating that the clinical spectrum of MIRAGE syndrome varies among subjects, accumulation of variant data and clarification of the genotype–phenotype correlation is a requisite to improve medical management of MIRAGE syndrome. To date, ~40 cases of MIRAGE syndrome have been reported^1,2,4,7–18^. Although the identified variants are scattered throughout the entire coding sequence with missense variants being most frequent, in vitro functional assays have revealed the consistent growth restriction phenotype of mutated SAMD9 protein. This suggests that the variant-site-based genotype–phenotype correlation has not been well determined for MIRAGE syndrome, although those who have a variant in an arginine residue have been reported to have more severe clinical presentations than those possessing non-arginine residues^2,7^. Thus, the accumulation of knowledge regarding novel variants and their associated clinical features is essential to better understand the variant-site-based genotype–phenotype correlation in MIRAGE syndrome.

In addition to the effects of variant site on the clinical course of MIRAGE syndrome, there is evidence of the clinical impact of additional genetic signatures in MIRAGE syndrome, which includes the loss of chromosome 7 on which the *SAMD9* gene variant responsible for MIRAGE syndrome is present^1,2^. Many patients develop transient anemia and thrombocytopenia, but become susceptible to the development of MDS when hematopoietic cells acquire monosomy 7 as an aneuploidy adaptive mechanism to protect cells from growth restriction caused by the MIRAGE variant. In this case, MDS did not develop at the age of 2, which was associated with the absence of monosomy 7. However, the karyotype was only evaluated during the neonatal period, and repeated karyotype analysis is required because monosomy 7 has been suggested to be acquired with age. In addition, a loss-of-function variant in the same allele in which a gain-of-function variant occurs has been demonstrated to rescue the growth restricting phenotype of the MIRAGE variant^2,11^, and the presence of a “reversion variant” may be associated with improvement or complete resolution of hematological manifestations, including MDS^18^. There are also familial cases who harbor a gain-of-function variant at low levels in cis with a more predominant null variant and do not present any manifestations of a MIRAGE phenotype but do have family members who show a MIRAGE phenotype because of the lack of null variant^15,17^. For example, Roucher-Boulez et al.^17^ recently reported a familial case of MIRAGE syndrome born to an asymptomatic mother carrying the same MIRAGE variant, which is likely caused by the occurrence of the in cis somatic reversion variant in the mother. The fact that the phenotype for patients with gain-of-function variants in the *SAMD9* gene ranges from apparently asymptomatic carriers, isolated bone marrow involvement, to a severe multisystem syndrome such as MIRAGE syndrome is likely due to multiple factors, including somatic reversion variants in various tissues in different stages of embryological development and postnatal life. Other levels of genetic modification in MIRAGE syndrome are characterized by the acquisition of uniparental disomy of the long arm of chromosome 7, which has been suggested to be protective against the development of MDS^9^. As these genetic modifications greatly impact the clinical course of MIRAGE syndrome, early genetic diagnosis together with the confirmation of age-dependent additional genetic features will help predict the clinical characteristics of patients and enable early and appropriate intervention.

In summary, we described the clinical characteristics of a patient with MIRAGE syndrome possessing a novel variant in the *SAMD9* gene. Although functional assays of the mutated protein and evaluations of genetic modifications are lacking, we believe that this case will further our knowledge regarding the genetic and clinical aspects of MIRAGE syndrome.

## Data Availability

The relevant data from this Data Report are hosted at the Human Genome Variation Database at 10.6084/m9.figshare.hgv.2811.
